# Constructing brain functional network by Adversarial Temporal-Spatial Aligned Transformer for early AD analysis

**DOI:** 10.3389/fnins.2022.1087176

**Published:** 2022-11-28

**Authors:** Qiankun Zuo, Libin Lu, Lin Wang, Jiahui Zuo, Tao Ouyang

**Affiliations:** ^1^School of Information Engineering, Hubei University of Economics, Wuhan, China; ^2^CAS Key Laboratory of Human-Machine Intelligence-Synergy Systems, Shenzhen Institutes of Advanced Technology, Chinese Academy of Sciences, and the SIAT Branch, Shenzhen Institute of Artificial Intelligence and Robotics for Society, Shenzhen, China; ^3^School of Mathematics and Computer Science, Wuhan Polytechnic University, Wuhan, China; ^4^Guangdong-Hong Kong-Macau Joint Laboratory of Human-Machine Intelligence-Synergy Systems, Shenzhen, China; ^5^State Key Laboratory of Petroleum Resource and Prospecting, and Unconventional Petroleum Research Institute, China University of Petroleum, Beijing, China; ^6^State Key Laboratory of Geomechanics and Geotechnical Engineering, Institute of Rock and Soil Mechanics, Chinese Academy of Sciences, Wuhan, China

**Keywords:** functional brain connectivity, temporal-spatial transformer alignment, generative adversarial learning, graph convolutional network, early Alzheimer's disease

## Abstract

**Introduction:**

The brain functional network can describe the spontaneous activity of nerve cells and reveal the subtle abnormal changes associated with brain disease. It has been widely used for analyzing early Alzheimer's disease (AD) and exploring pathological mechanisms. However, the current methods of constructing functional connectivity networks from functional magnetic resonance imaging (fMRI) heavily depend on the software toolboxes, which may lead to errors in connection strength estimation and bad performance in disease analysis because of many subjective settings.

**Methods:**

To solve this problem, in this paper, a novel Adversarial Temporal-Spatial Aligned Transformer (ATAT) model is proposed to automatically map 4D fMRI into functional connectivity network for early AD analysis. By incorporating the volume and location of anatomical brain regions, the region-guided feature learning network can roughly focus on local features for each brain region. Also, the spatial-temporal aligned transformer network is developed to adaptively adjust boundary features of adjacent regions and capture global functional connectivity patterns of distant regions. Furthermore, a multi-channel temporal discriminator is devised to distinguish the joint distributions of the multi-region time series from the generator and the real sample.

**Results:**

Experimental results on the Alzheimer's Disease Neuroimaging Initiative (ADNI) proved the effectiveness and superior performance of the proposed model in early AD prediction and progression analysis.

**Discussion:**

To verify the reliability of the proposed model, the detected important ROIs are compared with clinical studies and show partial consistency. Furthermore, the most significant altered connectivity reflects the main characteristics associated with AD.

**Conclusion:**

Generally, the proposed ATAT provides a new perspective in constructing functional connectivity networks and is able to evaluate the disease-related changing characteristics at different stages for neuroscience exploration and clinical disease analysis.

## 1. Introduction

Early Alzheimer's disease (AD) includes the following three successive stages: significant memory concern (SMC), early mild cognitive impairment (EMCI), and late mild cognitive impairment (LMCI). AD is a common long-term neurological disorder in the elderly, which is generally connected with the gradual decline in understanding, judgment, memory, and executive ability until complete loss. AD is known as the leading cause of death among old people worldwide (Zhang et al., 2022), and its great harmfulness brings heavy psychological pressure and economic burden to the families of patients. According to literature (Derby, 2020), the number of people suffering from AD and other dementias in the world currently exceeds 50 million, and the aging population further aggravates the rise of the patient population. However, there is no consensus on the pathological mechanism (Yuzwa et al., 2008; Diplas et al., 2018), and many pharmaceutical companies had tried and failed to develop effective drugs to cure AD. Therefore, early detection and timely intervention for AD are the only possible way in slowing down or preventing disease deterioration (Jack et al., 2013). The development of neuroimaging has made the use of non-invasive AD study become the mainstream of current research because of no side effects on patients (Wang et al., 2018c; Grassi et al., 2019; Yu et al., 2021; Alvi et al., 2022; Lei et al., 2022; You et al., 2022). It is very promising for the scientific community to develop effective methods to detect brain disease and assist clinical treatment from medical imaging data (Wang et al., 2022).

The brain functional network (BFN) derived from functional Magnetic Resonance Imaging (fMRI) describes the functional interactions among spatially distributed brain regions. Brain science indicates that abnormal functional connectivity always appears at the early stage of AD (Berron et al., 2020). The BFN can give a universal understanding of neurological symptoms and unravel the pathogenesis of cognitive diseases. As mentioned in Yu et al. (2020) and Zuo et al. (2021a), the whole brain is divided into several Region-of-Interests (ROIs) according to the anatomical template. The BFN is modeled as a graph, where each node represents the ROI and each edge represents the functional connection strength between paired ROIs. The conventional method is to use a software toolbox to construct functional connectivity (FC) and then extract effective features for disease diagnosis. For example, Kabbara et al. (2018) investigated the abnormal hub patterns associated with patients' cognitive performance by applying graph-theory analysis on the constructed functional connectivity. This work preserved the topological structure and gained better evaluation performance than the feature extraction algorithm (Wang et al., 2017; Zuo et al., 2021b; Yu et al., 2022) in Euclidean space. Considering the complexity of brain neural activities and noisy data preprocessed from the raw fMRI, it is significant for clinicians to investigate more advanced methods for modeling effective BFNs in early AD analysis.

Brain functional network construction by using time series can be divided into two classes: static-based method, and dynamic-based method. The former utilized the whole brain time series of fMRI to bridge links between ROIs for AD analysis. The direct way of constructing a brain functional network is to compute the person's correlation (PC) between any paired brain regions (Wang et al.,  2007). To reduce the possible impact of adjacent ROIs, Fransson and Marrelec (2008) employed partial correlations to handle this problem and achieved good performance in characterizing the changes of the default mode network associated with the disease. But the calculation of an inverse matrix usually comes up with multiple solutions, so researchers adopted certain constraints on the partial correlation estimation for a stable solution. For example, the matrix-regularized network was encoded as modularity prior to optimizing sparse brain network and they (Qiao et al., 2016) discovered potential biomarkers for personalized diagnosis. The latter method benefits the temporal changes of brain functional connectivity for capturing subtle transient neural abnormalities and has recently been a hot spot in neurological disease analysis. The direct approach is to generate a sequence of functional networks and designed a fused learning algorithm to jointly estimate the temporal network for early MCI detection (Wee et al., 2016). Furthermore, the work in Gong et al. (2022) treated the functional time series and functional connectivity as the node features and edges respectively, and developed a graph convolutional network (GCN) based model to generate multiple brain networks for characterizing brain temporal community by setting six-time sliding steps. To address the noisy problem of limited volumes in a sliding window, Zhou et al. (2018) proposed a matrix-regularized learning framework to learn sparse and modular high-order connectivity features for MCI classification. Although many studies have been conducted in BFN construction, they mainly rely on some specific preprocessing in the software toolboxes to obtain temporal features of each ROI. The drawbacks lie in two fields: one is that the multiple parameter settings may lead to different errors from person to person, and another is that a series of processes can consume much time and fall far away from the goal of clinical application.

Recently, data-driven models are capable of mining effective common characteristics from noisy data. It has been widely applied in various fields of medical image analysis, such as disease severe assessment (Wang et al., 2020c), lesion area segmentation (Hong et al., 2022b), health assessment (Wang et al., 2018b), disease detection (Wang et al., 2018a; Yang et al., 2022), image reconstruction (Hu et al., 2020b). To improve disease analysis performance, many advanced machine learning algorithms are designed to extract discriminative and robust features (Zeng et al., 2017; Lei et al., 2018; Hong et al., 2019; Wang et al., 2020b). Compared with the classification performance of traditional Convolutional Neural Networks (CNN) (Wang S.-Q. et al., 2015), the 3D Convolutional Neural Network (C3D) is good at capturing the local spatial features in a three-dimensional volume and has been successfully applied on the cross-modal image synthesis (Hu et al., 2020a) and disease recognition (Wang et al., 2020a). Moreover, the transformer network (Jiang et al., 2021) can model the global relationship between distant sub-patch regions. The ROI-based features can be learned by C3D and transformer in sequence from 4D fMRI data. Besides, Generative adversarial networks (GANs) are regarded as a special case of variational inference (Mo and Wang, 2009; Wang, 2009) and demonstrates impressive performance in matching generated data distributions. The obvious evidence is the success in generating cross-modal medical images (Hu et al., 2019, 2021) and domain adaptation segmentation (Hong et al., 2022a). It can be used as a regularizer to constrain the representation learning for stable and generalizable disease analysis.

Inspired by the above observations, in this paper, a novel Adversarial Temporal-Spatial Aligned Transformer (ATAT) model is proposed to automatically learn brain functional networks from 4D fMRI for detecting early AD. The constructed brain functional networks are also analyzed to identify important ROIs and abnormal connections. The main contributions of this work are as follows: (1) The region-sequence aligned generator (RAG) is developed to first learn rough ROI-based features by incorporating the brain anatomical information, then finely adjust the boundary features of adjacent ROIs to generate ROI time series and connectivity features. It greatly enhances the ROI time series learning and fully explores the spatial-temporal characteristics and connectivity information among the whole brain. (2) The multi-channel temporal discriminator is designed to constrain the learned ROI time series with the empirical samples. It regularizes the generator optimization and makes the connectivity feature more robust. (3) Experimental classification results prove the effectiveness of our model, and the discovered important ROIs and abnormal connections may be potential biomarkers for early AD diagnosis or treatment.

The rest of this article is organized as follows. Section 2 describes the novel proposed ATAT model for brain functional network construction. The experimental settings and prediction results with competing methods are presented in Section 3. In the Section 4, the reliability and limitations of this work are discussed. Finally, the Section 5 summarizes the main remarks of this paper.

## 2. Materials and methods

The proposed model includes three main parts, such as (1) data preprocessing, (2) architecture of the proposed model, and (3) objective functions for optimization.

### 2.1. Data description and preprocessing

The experimental data comes from the public Alzheimer's Disease Neuroimaging Initiative (ADNI-3). A total of 330 subjects with functional Magnetic resonance (fMRI) were downloaded from the website^1^, including 86 Normal Control (NC), and three successive stages of early AD (i.e., 82 SMC, 86 EMCI, 76 LMCI). The fMRI data is acquired under the 3.0 Tesla machine. The detailed scanning parameters for fMRI are as follows: the imaging resolution ranges from 2.5 to 3.75 mm along *X* and *Y* dimensional direction, the imaging slice thickness is between 2.5 and 3.4 mm; the time of repetition (TR) ranges from 0.607 to 3.0 s, and the time of echo (TE) value is in the range of 30 to 32 ms. The recording time length is about 10 min. The mean age of NC, SMC, EMCI, and LMCI is 74.4, 76.1, 75.7, and 75.8, respectively. The gender is roughly the same in each category.

The fMRI data is preprocessed by the software toolbox GRETNA (Wang J. et al., 2015), which contains about six procedures for constructing ROI-based time series. Each 4D fMRI data is processed by balancing magnetization equilibrium, removing head-motion artifacts, normalizing spatial space, smoothing, and filtering (0.01*Hz* ≤ *f* ≤ 0.08*Hz*). Finally, the automated anatomical labeling (AAL) atlas (Tzourio-Mazoyer et al., 2002) warps the preprocessed image to 90 non-overlapping spatial ROIs, and the final functional features with the size 90 × 187 are obtained as the truth samples. Meanwhile, the empirical functional connectivity is estimated by calculating the Pearson correlation coefficients between paired ROI time series, and this procedure can generate a 90 × 90 correlation matrix for each subject.

### 2.2. Architecture

**Figure 1:**
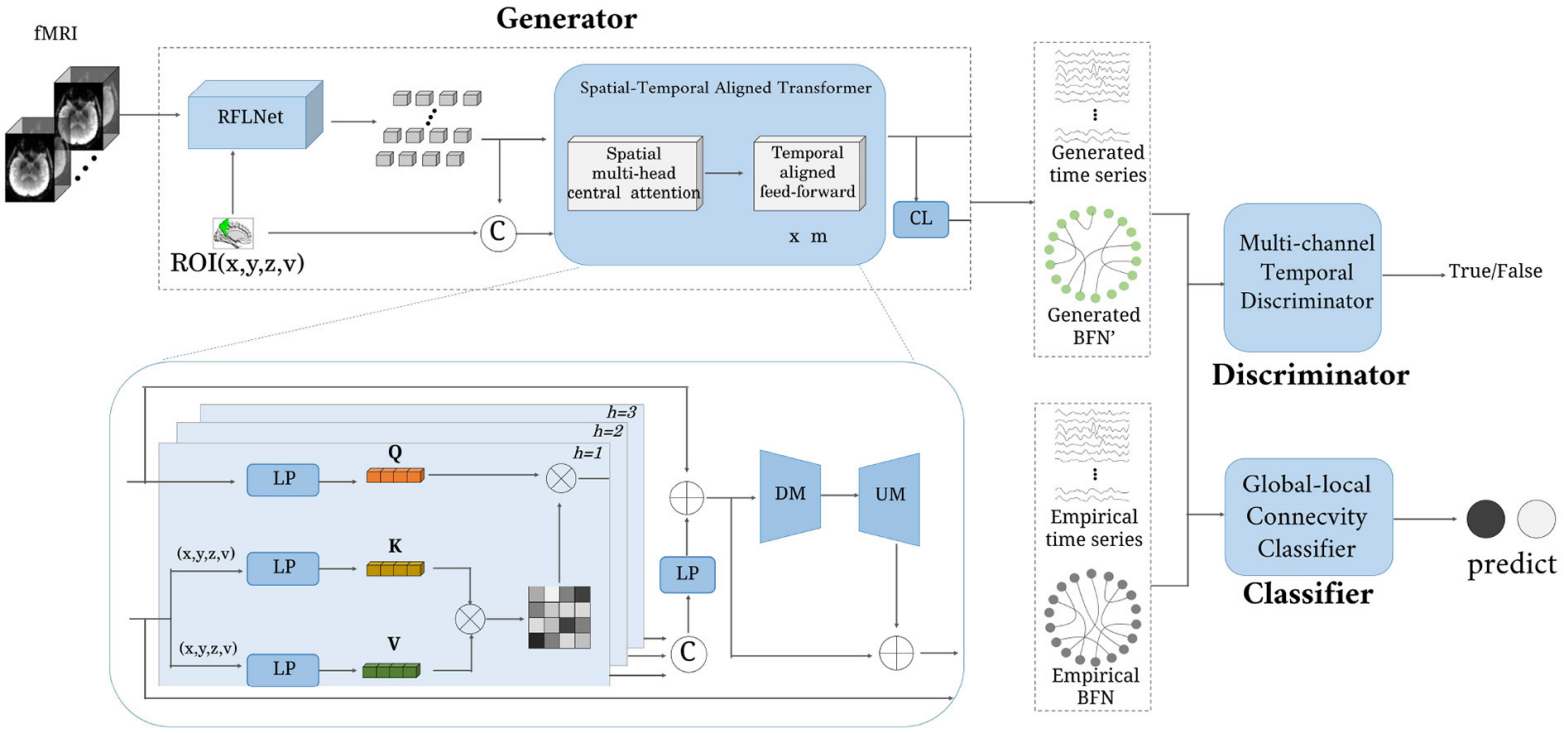
The framework of the proposed model. It consists of three parts: generator, discriminator, and classifier. The input is a four-dimensional fMRI, and the output is a brain functional network.

The architecture of the proposed ATAT is shown in Figure 1. It contains three parts: the region-sequence aligned generator (RAG), the multi-channel temporal discriminator (MTD), and the global-local connectivity classifier (GCC). The RAG includes a region-guided feature learning network(RFLNet), and a spatial-temporal aligned transformer (SAT), which transforms the 4D fMRI into ROI time series and brain functional network. Firstly, the raw fMRI data is first sent to the RFLNet for rough ROI-based feature extraction, and the SAT is utilized to finely adjust the feature for adjacent ROIs and align the global temporal correlation between any paired ROIs. Meanwhile, the obtained ROI time series is linearly transformed into brain functional networks through the connectivity learning (CL) network. After that, the generated ROI time series is constrained with the real sample distribution by the MTD. Finally, both ROI time series and brain functional networks are sent to the GCC for disease prediction. There are five objective functions in the model's optimization, including generator loss, discriminator loss, reconstruction loss, classifier loss, and regularized loss.

#### 2.2.1. Region-sequence aligned generator

##### 2.2.1.1. Region-guided feature learning network

**Figure 2:**
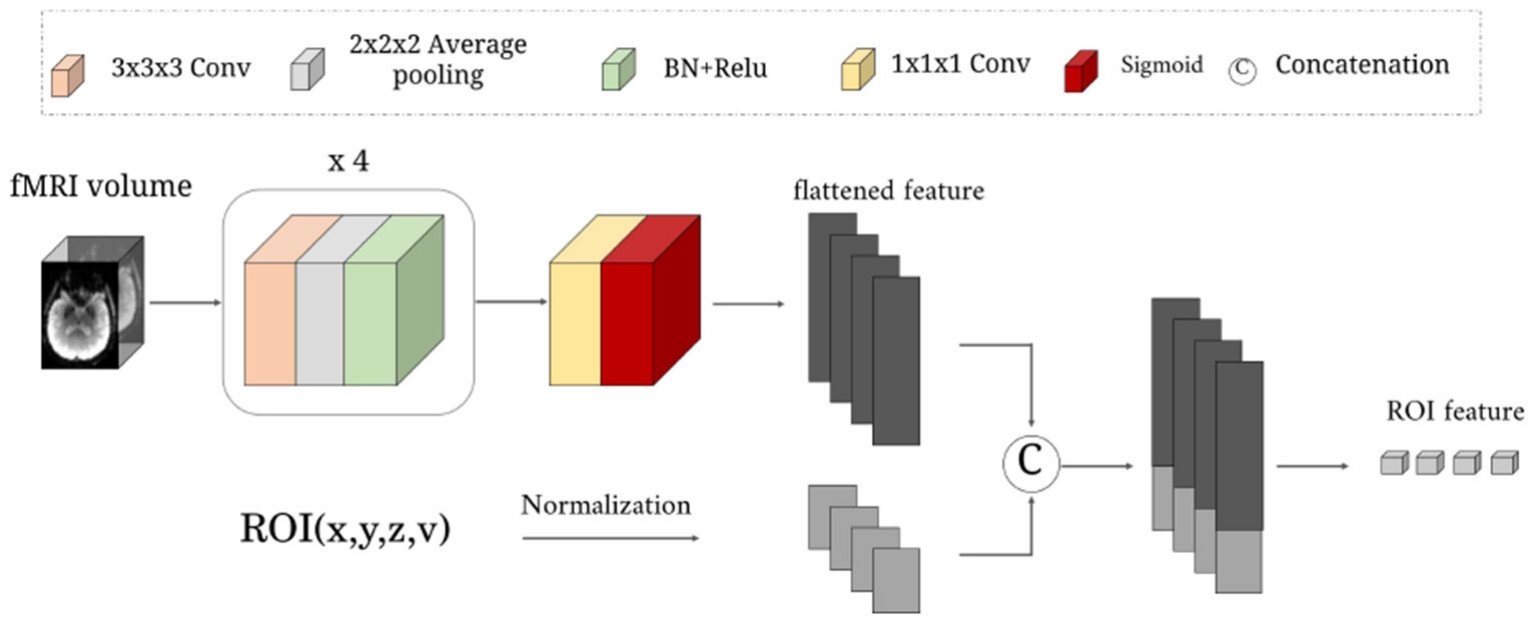
The detailed structure of the RFLNet. The input is an example of three-dimensional fMRI volume with the size 64 × 64 × 48, and the ROI information of the anatomical atlas with the size *N* × 4. The FRFNet outputs the initial feature for each ROI.

As illustrated in Figure 2. This network learns a rough mapping from the raw 4D fMRI to ROI-based time series by introducing the position and volume of the brain anatomical regions. The size of input data *X* is 64 × 64 × 48 × 187. It first passes through four blocks with three successive layers: 3 × 3 × 3 convolutional layers with 1-stride, 2 × 2 × 2 average pooling layer with 2-stride, and a combination layer of *batch normalization*(*BN*)+*ReLu* activation. The channel number of the above four convolutional layers are 8, 16, 32, 64. Then one 1 × 1 × 1 convolutional layer with 1-stride is used to increase the channels for matching the *N* ROIs, followed by a *sigmoid* activation function.

Next, we normalize the central location (*x, y, z*) and volume (*v*) of *N* anatomical ROIs to constrain the brain region information in the range 0 − 1. Finally, the (*x, y, z, v*) of *N* ROIs are treated as ROI embeddings, which are concatenated with the flattened feature of *sigmoid* layer output, which is sent to a one-layer linear projection (LP) layer for generating rough ROI features. The rough ROI feature can be expressed as:


(1) $$\begin{array}{l} F_{1}=\text{R}FLNe\text{t}\left(X,x,y,z,v\right) \end{array}$$

here, the *X* is the four dimensional volume data fMRI; *x, y, z, v*∈ℝ^*N*×1^; $F_{1}\in {\mathbb{R}}^{N\times q}$, the *RFLNet* is a combination of several convolution and pooling operations.

##### 2.2.1.2. Spatial-temporal aligned transformer

To learn more fined ROI temporal features, the spatial-temporal aligned transformer module is designed to recalibrate boundary ROI features and time sequence variations. It splits into two parts: the spatial multi-head central attention (SMCA) and the temporal aligned feed-forward (TAFF). Every ROI is regarded as a token. The rough ROI feature is first sent to three parallel LP layers to get query (**Q**), key (**K**), and value (**V**). Note that, the calculation of **K** and **V** needs to consider the ROI embeddings. The formulas can be defined as:


(2) $$\begin{array}{l} \text{Q}=LP\left(F_{1}\right),\;\text{K}=LP\left(F_{1}||x||y||z||v\right),\;\text{V}=LP\left(F_{1}||x||y||z||v\right) \end{array}$$

where, || means the concatenation operation. Then **Q**, **K**, **V**∈ℝ^*N*×*q*^ are separated into *h* heads. Each head of token (i.e., **Q**_*i*_, **K**_*i*_, **V**_*i*_) has the dimensional size *q*/*h*. Taking one head as an example, the central attention (CA) can be expressed:


(3) $$CA_{i}=Softmax(Q_{i}K_{i}^{T}/\sqrt{q/h})V_{i}$$

here, *i* means the index of *h* heads. The output of the spatial multi-head central-attention module is the concatenation of all heads and then with an LP layer (including residual mapping and layer normalization). It can be defined as:


(4) $$\begin{array}{l} SMCA=LP\left(CA_{1}||CA_{2}||...||CA_{h}\right)+F_{1} \end{array}$$

The *SMCA* has the size *N*×*q*.

Next, the TAFF module adjusts the temporal characteristics through the down mapping (DM) and up mapping (UM) layers and reduces the potential noise effect. The DM layer reduces the dimensional of *SMCA* from *q* to *q*/2, and the UM layer recovers the feature's dimension. Finally, the output of the TAFF module can be defined as:


(5) $$\begin{array}{l} F_{g}=UM\left(DM\left(SMCA\right)\right)+SMCA \end{array}$$

where, *F*_*g*_ is the generated ROI time series with the size *N*×*q*.

To learn an effective brain functional network *A*_*g*_, we first compute the Euclidean distance between any pair of ROI features and then apply a mapping matrix to it for similarity adjustment. Finally, a Gaussian kernel is introduced to learn non-linear projection for precise connectivity estimation. The formula can be defined as:


(6) $$\begin{array}{l} A_{g}\left(i,j\right)=exp\left(-\frac{{\left(F_{g}^{i}-F_{g}^{j}\right)}^{2}W}{2\sigma^{2}}\right) \end{array}$$

here, *A*_*g*_(*i, j*) represents the functional connectivity between pairwise ROIs. $F_{g}^{i}\in {\mathbb{R}}^{1\times q}$ means the *i*th ROI time series. *W*∈ℝ^*q*×*q*^ is time series transformable matrix. σ is the bandwidth of the Gaussian kernel, controlling the sparsity with the default value 2.

#### 2.2.2. Multi-channel temporal discriminator

**Figure 3:**
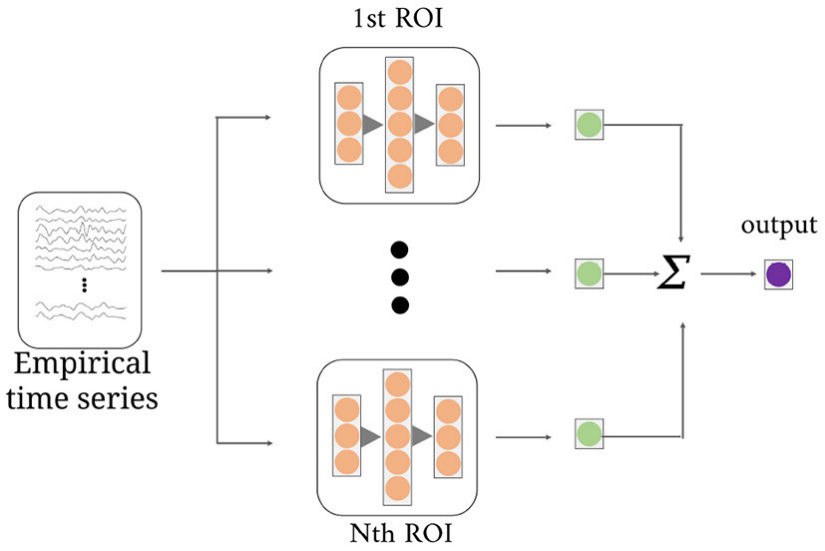
The structure of the multi-channel temporal discriminator. It accepts the empirical time series or the generated time series, and outputs the average discriminant result for distribution constraints of all ROI time series.

As shown in Figure 3, the multi-channel temporal discriminator (MTD) is used to constrain the generated functional time series (*F*_*g*_) distribution consistent with the empirical functional time series (*F*_*e*_). The *F*_*e*_ is computed from the software toolbox, which is treated as the true sample. The structure of MTD consists of *N* parallel networks, containing three linear projections with *q*/2, *q*, and *q*/2 neurons. Each MTD accepts *i*-th ROI time series and outputs one discriminate value. Averaging all the discriminate values is the final discriminate result.

#### 2.2.3. Global-local connectivity classifier

**Figure 4:**
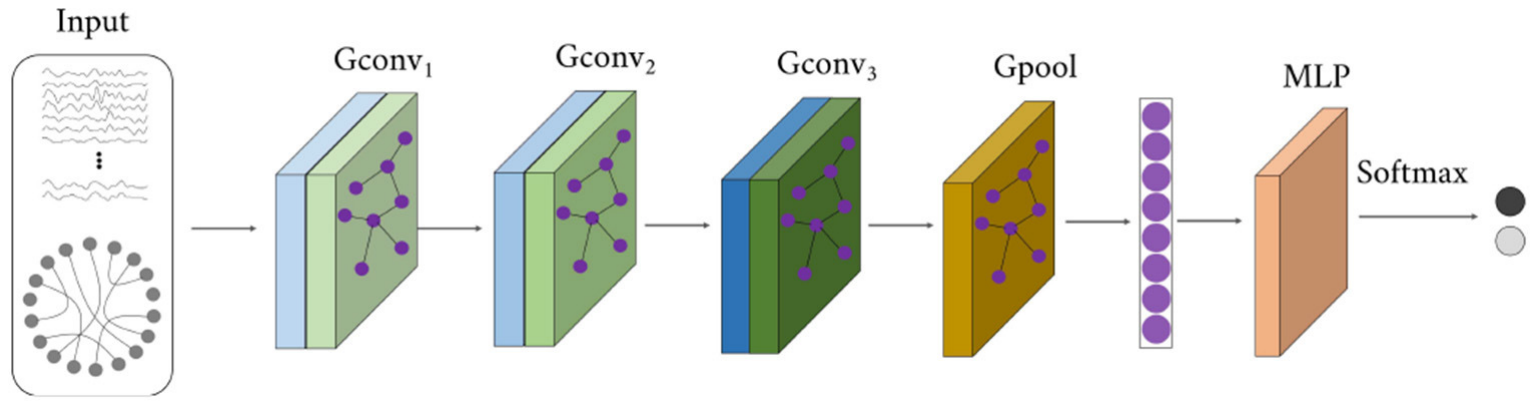
The structure of the global-local connectivity classifier.

The structure of the global-local connectivity classifier (GCC) is illustrated in Figure 4. It accepts both functional time series (i.e., *F*_*e*_ or *F*_*g*_) and functional network (i.e., *A*_*e*_ or *A*_*g*_), outputs the disease label. A total of 5 layers are designed in the GCC, including three graph convolutional layers, one graph pooling layer, and one three-layer perceptron. It is based on the graph convolutional network. The first three layers (i.e., *Gconv*_1_, *Gconv*_2_, *Gconv*_3_) are utilized to diffuse global features and reduce the ROI feature dimension. The graph pooling layer (*Gpool*) is utilized to average features along the ROI feature dimension and get one value for each ROI. And the MLP layer learns a linear mapping to recognize the disease.

### 2.3. Objective functions

In this section, the five loss functions defined below are utilized to optimize the model for disease prediction and analysis. The reconstruction loss *L*_*rec*_ can constrain the generator and retain the empirical features *F*_*e*_, the generate loss *L*_*g*_ and discriminate loss *L*_*d*_ are combined to optimize the generator and discriminator, the classification loss *L*_*cls*_ and regularized loss *L*_*reg*_ are utilized to upgrade the parameters of CL network and GCC network. For the convenience of explanation, we make the following simplification: *G* means all the operations in the Region-sequence aligned generator, *D* means the multi-channel temporal discriminator, and *C* is the global-local connectivity classifier. The raw fMRI data *X* follows the distribution *P*_*fMRI*_, the *P*_*F*_*e*__ and *P*_*A*_*e*__ represent the empirical functional time series *F*_*e*_ and empirical BFN *A*_*e*_ distribution, respectively. *Y* is the truth label. These loss functions are defined as follows:


(7) $$\begin{array}{l} L_{rec}={𝔼}_{X\sim P_{fMRI},F_{e}\sim P_{F_{e}}}\left(\;||G\left(X\right)-F_{e}||\;\right) \end{array}$$

(8) $$\begin{array}{l} L_{g}={𝔼}_{X\sim P_{fMRI}}\left[\;{\left(1-D\left(G\left(X\right)\right)\right)}^{2}\;\right] \end{array}$$

(9) $$\begin{array}{l} L_{d}={𝔼}_{X\sim P_{fMRI}}\left[\;{\left(D\left(G\left(X\right)\right)\right)}^{2}\;\right]+{𝔼}_{F_{e}\sim P_{F_{e}}}\left[\;{\left(1-D\left(F_{e}\right)\right)}^{2}\;\right] \end{array}$$

(10) $$\begin{array}{l} L_{cls}={𝔼}_{X\sim P_{fMRI}}\left[-Y\cdot log\left(C\left(G\left(X\right)\right)\right)\right]\\ \;+{𝔼}_{A_{e}\sim P_{A_{e}},F_{e}\sim F_{A_{e}}}\left[-Y\cdot log\left(C\left(A_{e},F_{e}\right)\right)\right] \end{array}$$

(11) $$\begin{array}{l} L_{reg}=𝔼\left(\left||W|\right|\right) \end{array}$$

The hybrid cost of the proposed model is:


(12) $$\begin{array}{l} L_{all}=L_{rec}+L_{g}+L_{d}+L_{cls}+\lambda L_{reg} \end{array}$$

## 3. Experiments and results

### 3.1. Experimental setup

There are six binary classification tasks for the evaluation of the proposed model, including NC vs. SMC, NC vs. EMCI, NC vs. LMCI, SMC vs. EMCI, SMC vs. LMCI, and EMCI vs. LMCI. The evaluation metrics are Accuracy (ACC), Sensitive (SEN), Specificity (SPE), and F1-score. We repeated the 10 times experiment using the five-fold cross-validation on each binary classification and utilized the mean value metrics for the final prediction. To demonstrate our model's good ability in FBN construction, we introduce two classifiers [i.e., SVM (Suthaharan, 2016) and GCN (Kipf and Welling, 2016)] to compare the BFN constructed by ATAT and Empirical.

Our proposed model was implemented with the TensorFlow framework on Ubuntu18.04 and the GPU of NVIDIA GeForce RTX 3080 Ti. The parameters in the experiments are defined as follows: *N* = 90, *q* = 187, *h* = 11, *m* = 3, λ = 10^−5^. During the training, we first update the weights in the generator and the discriminator, then fix part of the generator and optimize the network of CL and GCC. The learning rate of the generator and the classifier were set to 0.0001, and for the discriminator, the learning rate is set to 0.0004. The Adam was adopted for training the proposed model with batch size 2.

### 3.2. Prediction results

**Figure 5:**
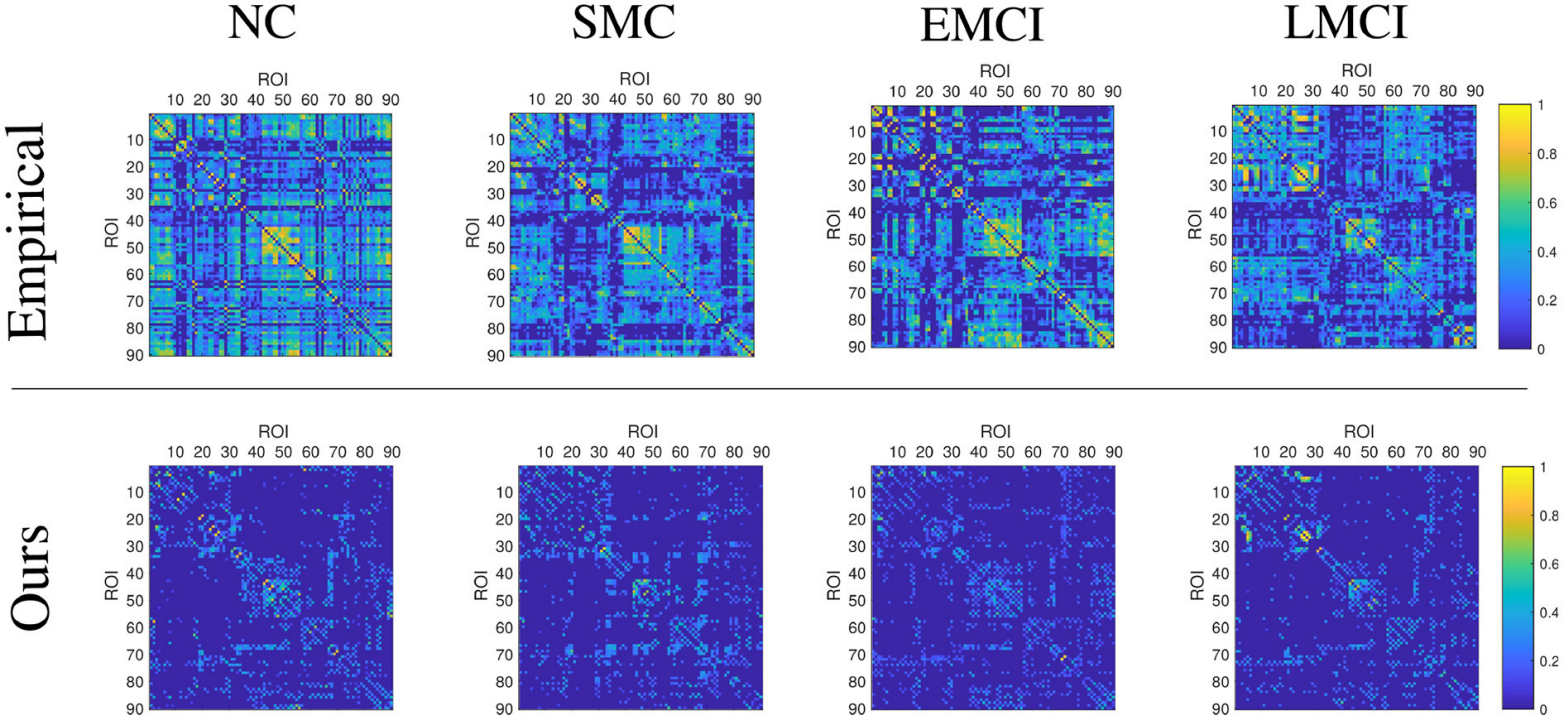
Display of brain functional network examples at different disease stages. The BFNs in the upper row are generated by the GRETNA toolbox, and the BFNs in the lower row are generated by the proposed model.

**Figure 6:**
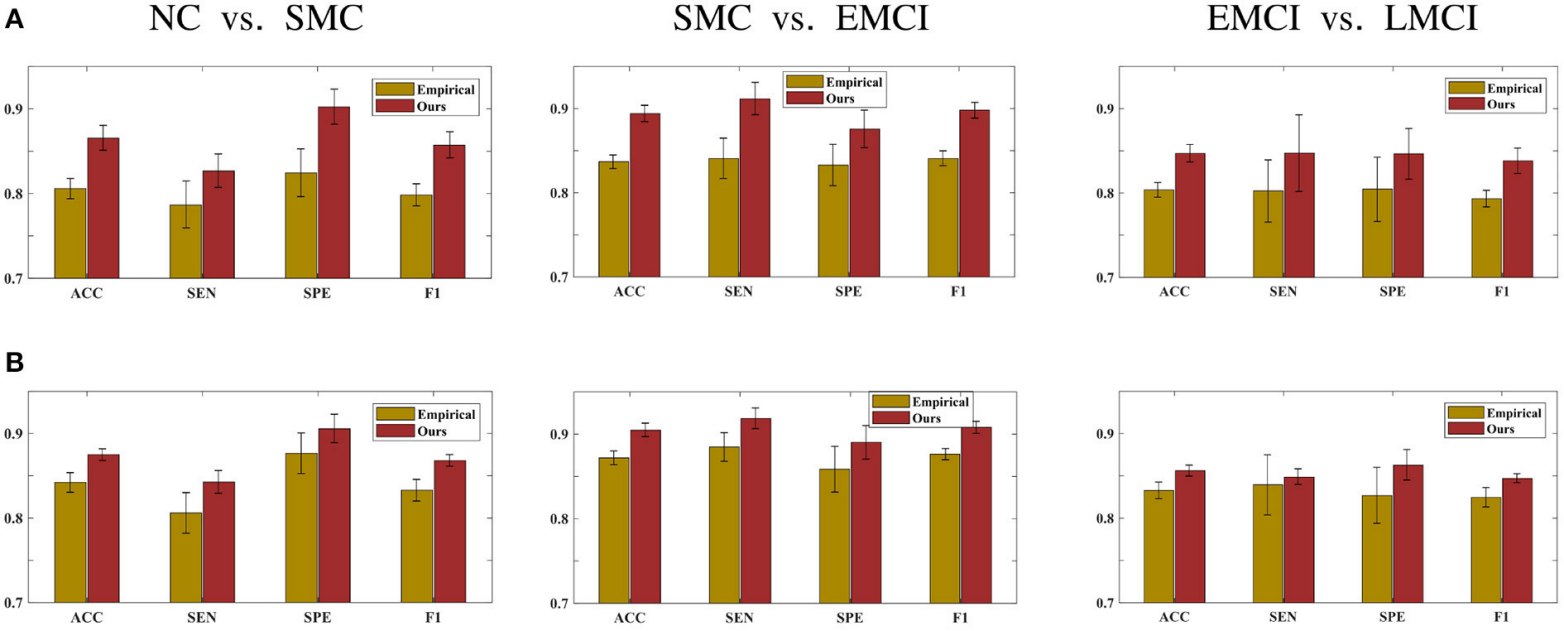
Prediction results of three scenarios tasks using **(A)** the SVM classifier and **(B)** the GCN classifier.

This section demonstrates the good performance of BFN constructed by the proposed model. As shown in Figure 5, the upper row shows the four stages of empirical FBN derived from the GRETNA, while the lower row displays the corresponding FBNs by the proposed model. The main connectivity patterns have been preserved and dense connections become sparse by comparing the empirical and ours. Figure 6 gives the classification result comparison in terms of three scenarios tasks. For the GCN classifier, the BFNs constructed by ours achieve the best prediction results with a mean ACC of 87.50%, a mean SEN of 84.26%, a mean SPE of 90.58% and a mean F1 of 86.81% in NC vs. SMC task; the mean values of SMC vs. EMCI are 90.47, 91.86, 89.02, and 90.80%; in EMCI vs. LMCI task, the predicted results are 85.61, 84.86, 86.27, and 84.70%. The standard error also shows the superior stability of the proposed model.

**Figure 7:**
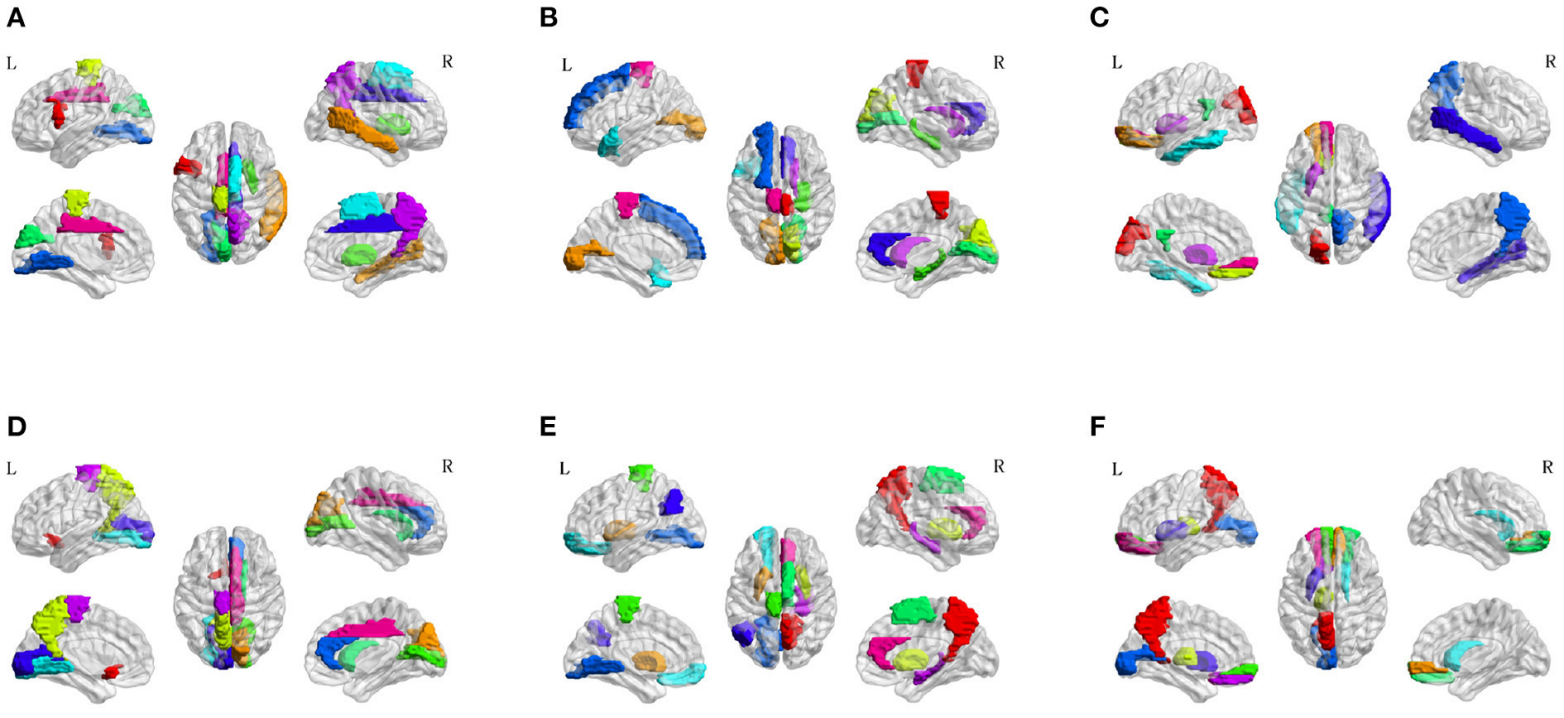
Spatial visualization of top 10 brain regions in the six classification scenarios. **(A)** NC vs. SMC. **(B)** NC vs. EMCI. **(C)** NC vs. LMCI. **(D)** SMC vs. EMCI. **(E)** SMC vs. LMCI. **(F)** EMCI vs. LMCI.

To investigate the potential AD-related ROIs, we shield one brain region and calculate the classification ACC as the effect of this ROI on AD progression. After sorting the ACCs in ascending order, the top 10 values are the most important ROI in the classification evaluation. As is shown in Figure 7, the spatial distribution of 10 important AD-related ROIs is displayed in lateral, medial, and dorsal views using the BrainNet Viewer (Xia et al., 2013). Specifically, the top 10 related ROIs are IFGoperc.L, MTG.R, PCL.L, PUT.R, CUN.L, SMA.R, LING.L, DCG.R, PCUN.R, DCG.L in NC vs. SMC classification scenario; The ten ROIs, including PCL.R, CAL.L, CUN.R, HIP.R, CAL.R, TPOsup.L, SFGdor.L, ACG.R, CAU.R, PCL.L, are important for NC vs. EMCI; also, the top 10 ROIs of NC vs. LMCI are SOG.L, ORBsup.L, REC.L, PUT.L, PCG.L, ITG.L, PCUN.R, MTG.R, PUT.L, ORBsupmed.L; For SMC vs. EMCI and SMC vs. LMCI classification, the important ROIs are OLF.L, CUN.R, PCUN.L, CAL.R, CAU.R, LING.L, ACG.R, CAL.L, PCL.L, DCG.R, and PCUN.R, PUT.L, PUT.R, PCL.L, SMA.R, ORBsup.L, LING.L, ANG.L, HIP.R, ACG.R, respectively; For EMCI vs. LMCI, the important ROIs are PCUN.L, ORBsupmed.R, THA.L, ORBsupmed.L, ORBsup.R, CAU.R, CAL.L, PUT.L, REC.L, ORBsup.L.

### 3.3. Brain network analysis

**Figure 8:**
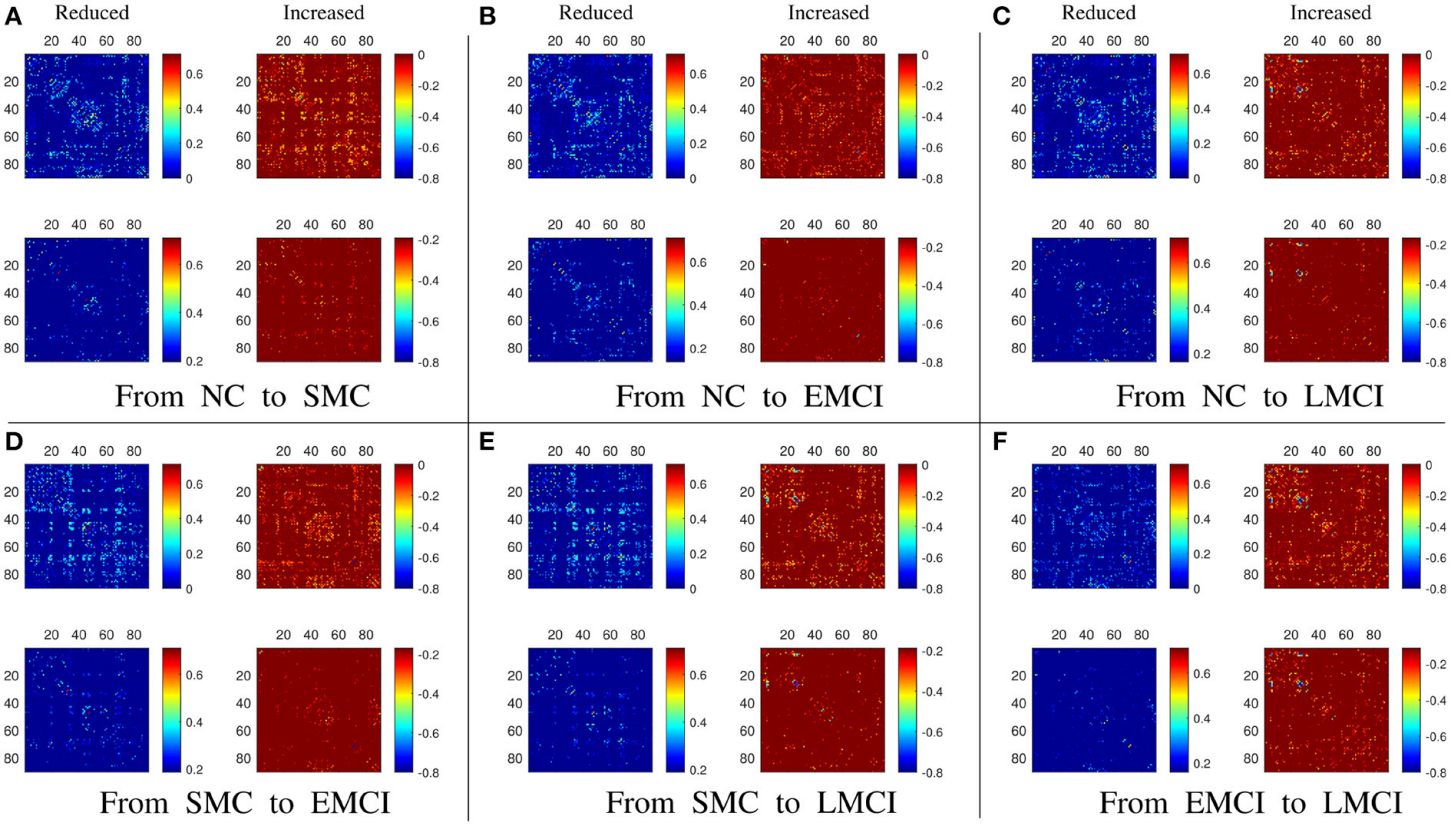
**(A–F)** The results of the altered functional connectivity estimated from the averaged BFNs between different groups (i.e., NC vs. SMC, NC vs. EMCI, NC vs. LMCI, SMC vs. EMCI, SMC vs. LMCI, EMCI vs. LMCI). In each subfigure, the upper row means the reduced and increased connections, the lower row shows the altered connections selected from the upper row with a threshold of 90% quantile value.

**Figure 9:**
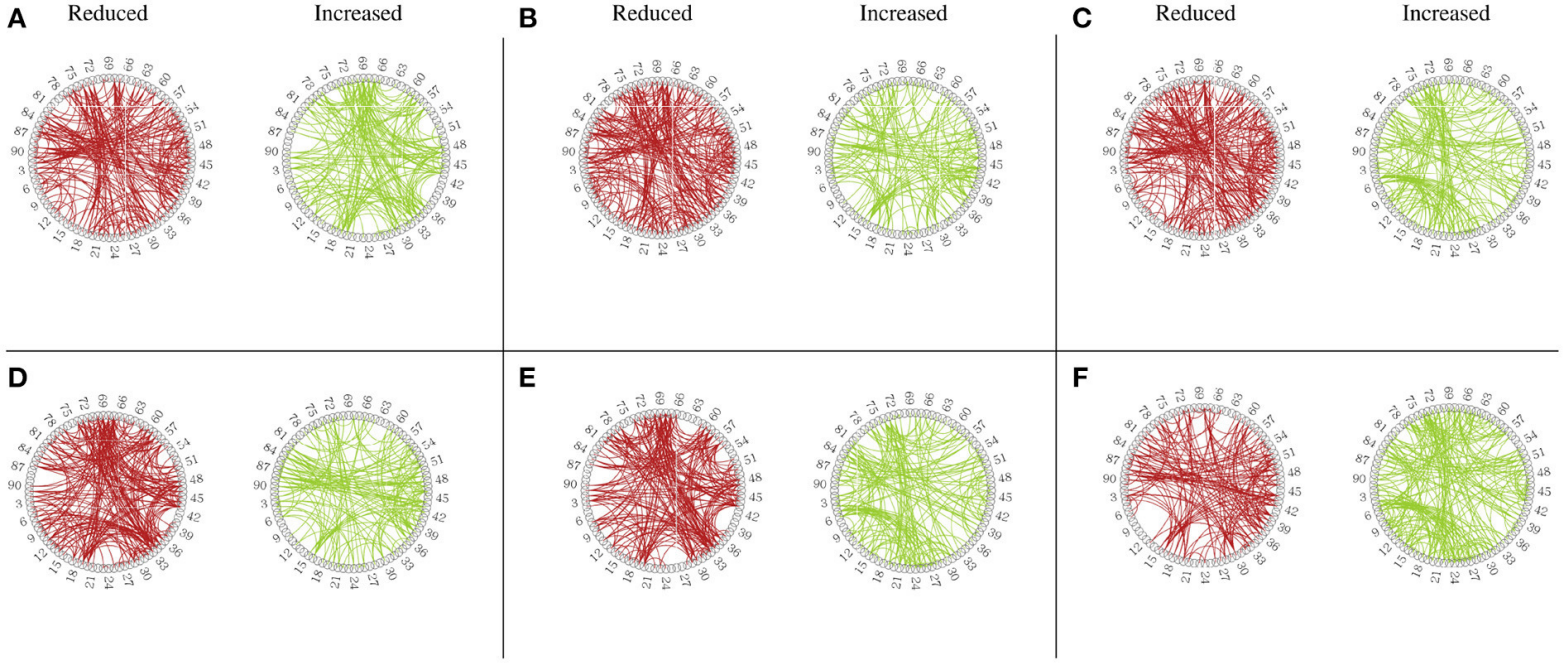
Circular graph of altered functional connectivity in MCI patients among 90 Anatomical Automatic Labeling (AAL) atlas regions. **(A)** From NC to SMC. **(B)** From NC to EMCI. **(C)** From NC to LMCI. **(D)** From SMC to EMCI. **(E)** From SMC to LMCI. **(F)** From EMCI to LMCI.

**Figure 10:**
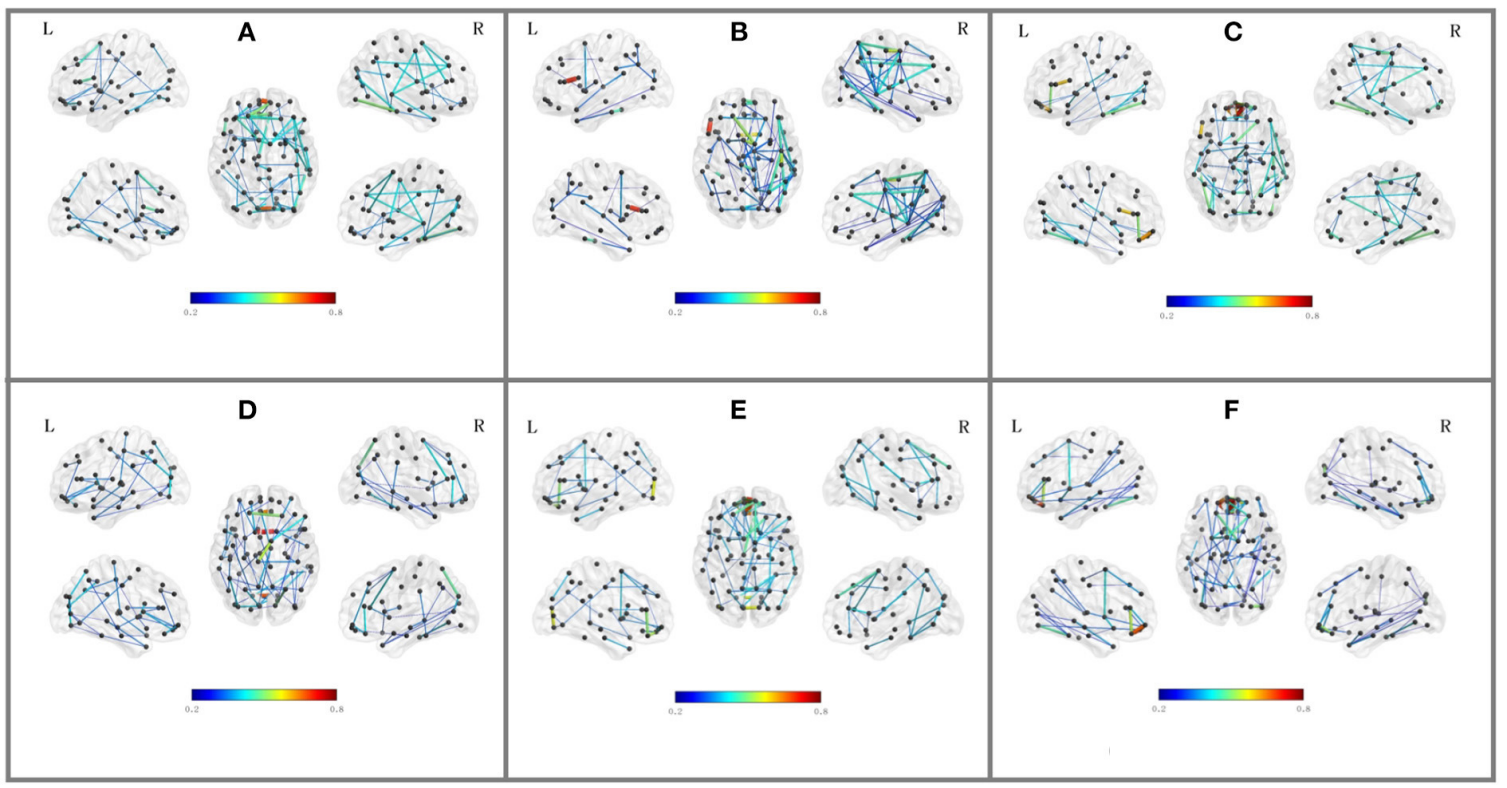
Top 2% altered functional connections in strength evaluation in the six classification scenarios. Each subfigure shares the same color bar, which means the absolute connection strength. **(A)** NC vs. SMC. **(B)** NC vs. EMCI. **(C)** NC vs. LMCI. **(D)** SMC vs. EMCI. **(E)** SMC vs. LMCI. **(F)** EMCI vs. LMCI.

**Figure 11:**
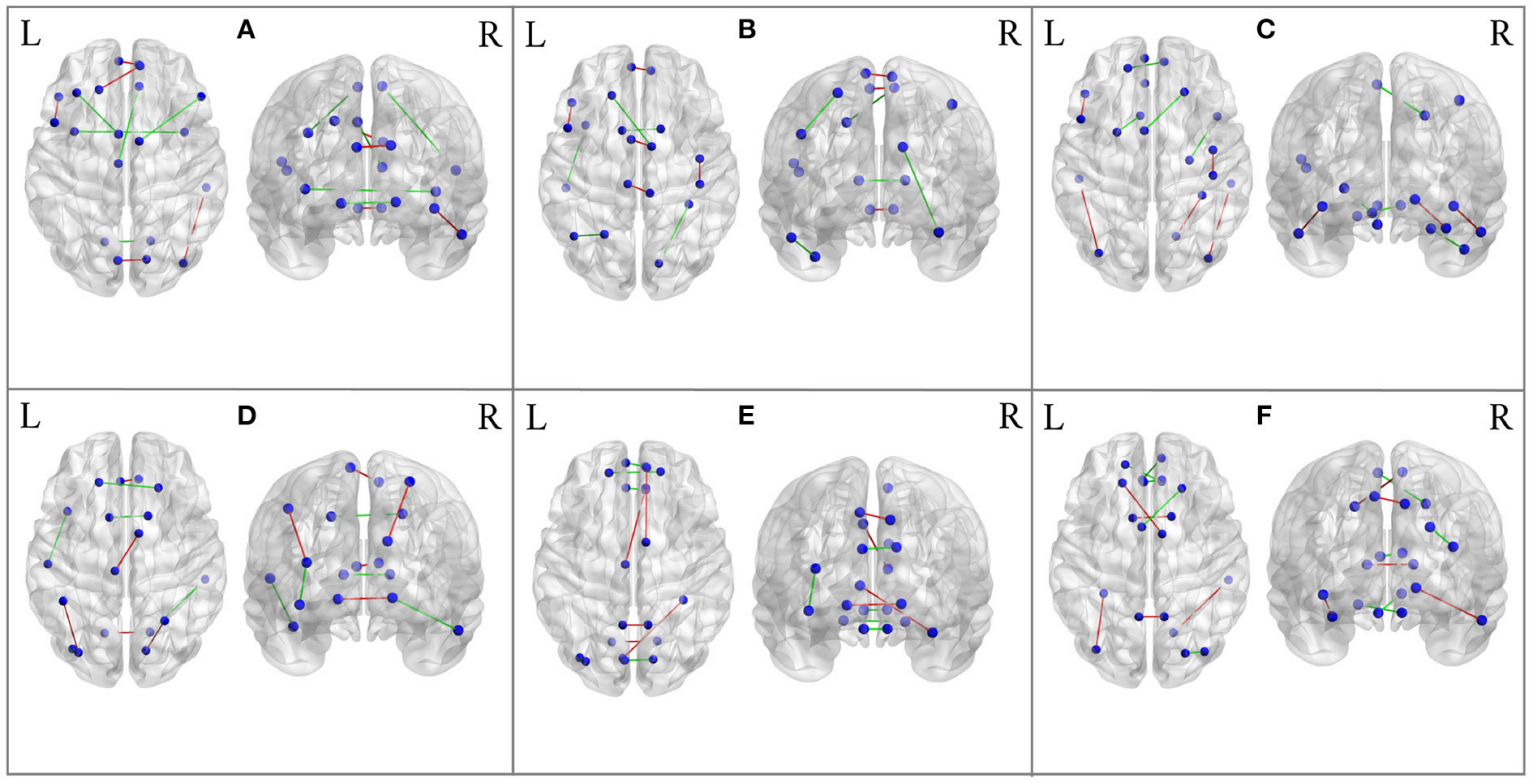
The most significant 5 reduced connections and 5 increased connections mapped on the AAL 90 template using the BrainNet Viewer software package. Blue color means the ROIs, red color means reduced connections, and green color means increased connections. **(A)** From NC to SMC. **(B)** From NC to EMCI. **(C)** From NC to LMCI. **(D)** From SMC to EMCI. **(E)** From SMC to LMCI. **(F)** From EMCI to LMCI.

Besides the prediction of different early AD stages, the other major purpose is to analyze the learned FBNs. After applying the ATAT model to each subject, we can obtain the mean FBN for each group of patients (i.e., NC, SMC, EMCI, and LMCI). To investigate the altered connectivity of FBN between different groups, we compute the difference of six paired scenarios as shown in Figure 8. In each subplot, reduced and increased connectivity can be observed between different paired groups. To analyze the significant connections, we set the 90% quantile value of the altered connectivity strength as the threshold. The pictures in the lower row of each subplot are the corresponding connectivity matrices by setting the threshold value. Figure 9 shows these significant connections in a circular graph. The number of reduced connections are 219, 263, 235, 251, 222, and 163 for NC vs. SMC, NC vs. EMCI, NC vs. LMCI, SMC vs. EMCI, SMC vs. LMCI, EMCI vs.LMCI, respectively; the corresponding number of increased connections are 183, 139, 166, 150, 179, 239. To show the main connectivity patterns in different classification scenarios, we select the top 2% largest altered connections (i.e., reduced, and increased). As shown in Figure 10, different connectivity patterns can be seen in different classification scenarios. Figure 11 depicts the top 5 reduced and top 5 increased connections in the axial and coronal view direction. The connectivity-related ROIs are listed in Tables 1, 2.

**Table 1:** The top 10 significant altered connections estimated from the generated FBNs in NC vs. SMC, NC vs. EMCI, NC vs. LMCI using AAL90 template (− means reduced connections, + means increased connections).

	**From NC to SMC**		**From NC to EMCI**		**From NC to LMCI**	
	**Indices**	**Names**	**Indices**	**Names**	**Indices**	**Names**
	11, 13	IFGoperc.L, IFGtriang.L	11, 13	IFGoperc.L, IFGtriang.L	11, 13	IFGoperc.L, IFGtriang.L
	3, 24	SFGdor.L, SFGmed.R	19, 20	SMA.L,SMA.R	48, 56	LING.R, FFG.R
-	25, 26	ORBsupmed.L, ORBsupmed.R	25, 26	ORBsupmed.L, ORBsupmed.R	2, 58	PreCG.R, PoCG.R
	45, 46	CUN.L, CUN.R	2, 58	PreCG.R, PoCG.R	53, 89	IOG.L, ITG.L
	54, 90	IOG.R, ITG.R	6 9, 70	PCL.L, PCL.R	54, 90	IOG.R, ITG.R
	7, 19	MFG.L, SMA.L	3, 20	SFGdor.L, SMA.R	4, 19	SFGdor.R, SMA.L
	14, 20	IFGtriang.R, SMA.R	46, 56	CUN.R, FFG.R	5, 26	ORBsup.L, ORBsupmed.R
+	29, 30	INS.L, INS.R	59, 65	SPG.L, ANG.L	25, 27	ORBsupmed.L, REC.L
	32, 33	ACG.R, DCG.L	71, 72	CAU.L, CAU.R	21, 73	OLF.L, PUT.L
	47, 48	LING.L, LING.R	87, 89	TPOmid.L, ITG.L	40, 88	PHG.R, TPOmid.R

**Table 2:** The top 10 significant altered connections estimated from the generated FBNs in SMC vs. EMCI, SMC vs. LMCI, EMCI vs. LMCI using AAL90 template (− means reduced connections, + means increased connections).

	**From SMC to EMCI**		**From SMC to LMCI**		**From EMCI to LMCI**	
	**Indices**	**Names**	**Indices**	**Names**	**Indices**	**Names**
	31, 32	ACG.L, ACG.R	20, 24	SMA.R, SFGmed.R	3, 20	SFGdor.L, SMA.R
	47, 48	LING.L, LING.R	32, 33	ACG.R, DCG.L	53, 55	IOG.L, FFG.L
-	46, 60	CUN.R, SPG.R	47, 48	LING.L, LING.R	67, 68	PCUN.L, PCUN.R
	51, 61	MOG.L, IPL.L	43, 56	CAL.L, FFG.R	71, 72	CAU.L, CAU.R
	20, 69	SMA.R, PCL.L	67, 68	PCUN.L, PCUN.R	48, 90	LING.R, ITG.R
	3, 4	SFGdor.L, SFGdor.R	5, 6	ORBsup.L, ORBsup.R	4, 19	SFGdor.R, SMA.L
	51, 53	MOG.L, IOG.L	25, 26	ORBsupmed.L, ORBsupmed.R	26, 27	ORBsupmed.R, REC.L
+	71, 72	CAU.L, CAU.R	27, 28	REC.L, REC.R	5, 28	ORBsup.L, REC.R
	81, 83	STG.L, TPOsup.L	45, 46	CUN.L, CUN.R	31, 32	ACG.L, ACG.R
	48, 90	LING.R, ITG.R	51, 53	MOG.L, IOG.L	50, 52	SOG.R, MOG.R

## 4. Discussion

### 4.1. Effect of the generator

**Figure 12:**
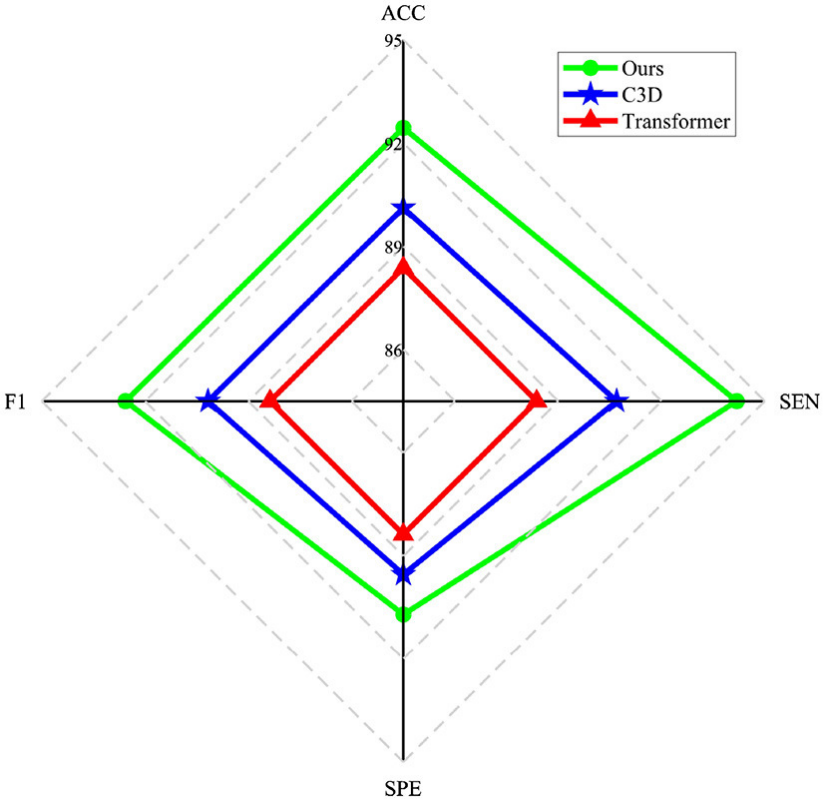
Influence of different generator structures on the classification performance.

**Figure 13:**
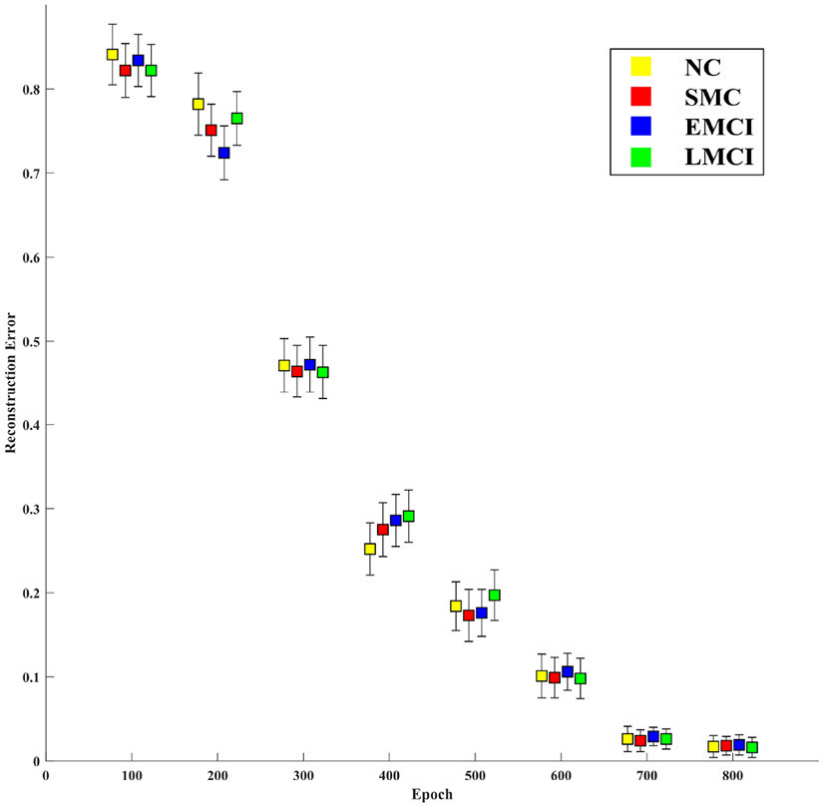
Mean absolute error between the empirical and generated time series over the training processes.

The main goal of the proposed model is to generate BFNs from 4D fMRI data. The modules in the generator play an important role in disease prediction and brain network analysis. To investigate the influence of the generator structure on the classification performance (i.e., NC vs. LMCI), we replace the RFLNet and the SAT modules with traditional C3D (Hong et al., 2020) and transformer (Jiang et al., 2021), respectively. In both cases, the anatomical ROI information is not included in the module. Figure 12 shows that either the C3D or Transformer network can degrade the prediction performance, and the traditional transformer network has a worse influence on classification than the C3D network. It may indicate the proposed RFLNet learns rough ROI-based features with a litter effect on the results, and the SAT network finely adjusts the adjacent ROI-based temporal features which may greatly influence the classification performance. Furthermore, the reconstructed error of the ROI time series is measured by the mean absolute error (MAE) metric. As shown in Figure 13, the divergence of the MAE for each disease (i.e., NC, SMC, EMCI, and LMCI) demonstrates the reliable results of the designed generator.

### 4.2. Comparison with related works

In the six classification scenarios, there are eight ROIs that overlap more than three times in the identified brain regions. These important ROIs are the orbital part of the superior frontal gyrus (ORBsup.L), the anterior cingulate and paracingulate gyri (ACG.R), the calcarine fissure and surrounding cortex (CAL.L), the lingual gyrus (LING.L), the precuneus (PCUN.R), the paracentral lobule (PCL.L), the caudate nucleus (CAU.R), the lenticular nucleus putamen (PUT.L). Most of these ROIs are consistent with the previous studies (Li et al., 2017; Yu et al., 2017; Ye et al., 2019), which demonstrates the strongly correlation with AD. In addition, the most significant altered connectivity (also called abnormal connections) related ROIs contain the identified eight brain regions. These listed ROIs in the table are mainly distributed in the frontal lobe, temporal lobe, and occipital lobe. The frontal lobe is located in the most anterior part of the cerebral hemispheres, accounting for the first 1/3 of the surface of the cerebral hemispheres. It is mainly related to higher mental activity, including physical activity control, language speaking, self-awareness, and emotional expression. The identified ROIs (i.e., SFGdor, ORBsup, IFGoperc, IFGtriang) associated with the superior frontal gyrus can be founded in Whitwell et al. (2007). The visual and language information is memorized by the temporal lobe, in which patients with AD showed abnormal levels of tau protein in the inferior temporal gyrus (Mormino et al., 2016). The occipital lobe participates in visual processing, for example, the lingual gyrus shows altered functional connectivity in AD patients (Skouras et al., 2019). In general, the derived important ROIs and abnormal connections by the proposed model can reflect the main characteristics associated with AD.

### 4.3. Limitations and future directions

Although the proposed model in the experiment has achieved good classification results and reliable connectivity analysis, there are two deficiencies in this work. One is that it only considers the binary classification tasks, which may not capture the continuously changing characteristics during the disease progression. We will conduct multi-class classification experiments to investigate the common changes at different stages of AD. Another limitation is that the dataset used in this study is relatively small. In the future study, we will increase the amount of data to validate the proposed model for brain disorder analysis by using other larger datasets [UK biobank (Sudlow et al., 2015), ABIDE (Heinsfeld et al., 2018)].

## 5. Conclusion

In this paper, we proposed a novel ATAT model to construct brain functional networks for early AD diagnosis and analysis. The three-player generative adversarial network is alternatively optimized and can learn effective functional connectivity features from 4D fMRI. By incorporating the brain anatomical information, the rough ROI features can be extracted by focusing on the local spatial information of individual brain region. Furthermore, the SAT module explores the temporal characteristics and connectivity information for finely adjusting the boundary features of adjacent ROIs. Meanwhile, the generated features from the region-sequence aligned generator are constrained by the adversarial loss and reconstruction loss. Compared to the empirical method, the brain functional networks constructed by the proposed model achieve higher classification performance. The identified important ROIs and abnormal connections may be the potential biomarkers for early AD diagnosis. Generally, our proposed model has the potential in constructing complex functional connectivity features and exploring abnormal functional connections for neurodegenerative diseases study.

## Data availability statement

The original contributions presented in the study are included in the article/supplementary material, further inquiries can be directed to the corresponding author/s.

## Author contributions

QZ designed the methodology, conducted the experiment, and wrote the manuscript. LW was the head of the funds of the paper and supervised and verified the reliability of the experimental results. LL provided suggestions for result visualization. JZ and TO reviewed and edited the manuscript. All authors read and approved the final manuscript.

## Funding

This work was supported in part by National Key R&D Program of China under grant 2020YFC2005803, International Partnership Program of Chinese Academy of Sciences under grant GJHZ2021132, and NSFC 62003331.

## Conflict of interest

The authors declare that the research was conducted in the absence of any commercial or financial relationships that could be construed as a potential conflict of interest.

## Publisher's note

All claims expressed in this article are solely those of the authors and do not necessarily represent those of their affiliated organizations, or those of the publisher, the editors and the reviewers. Any product that may be evaluated in this article, or claim that may be made by its manufacturer, is not guaranteed or endorsed by the publisher.

## Footnotes

^1^ http://adni.loni.usc.edu/
